# Recent developments in 2D MXene-based materials for next generation room temperature NO_2_ gas sensors

**DOI:** 10.1039/d3na00275f

**Published:** 2023-08-15

**Authors:** Sithara Radhakrishnan, Chandra Sekhar Rout

**Affiliations:** a Centre for Nano and Material Sciences, Jain (Deemed-to-be University) Jain Global Campus, Kanakapura Bangalore 562112 Karnataka India r.chandrasekhar@jainuniversity.ac.in csrout@gmail.com

## Abstract

MXenes with distinctive structures, good electrical conductivity and abundant functional groups have shown great potential in the fabrication of high performance gas sensors. Since the sensing mechanism of MXene-based gas sensors often involves a surface-dominant process, they can work at room temperature. In this regard, a significant amount of research has been carried out on MXene-based room temperature gas sensors and they can be viewed as one of the possible materials for NO_2_ sensing applications in the future. In this review, we focus on the most recent research and improvements in pure MXenes and their nanocomposites for NO_2_ gas sensing applications. First, we have explored the mechanisms involved in MXenes for NO_2_ gas sensing. Following that, other ways to tune the MXene sensing performance are investigated, including nanocomposite formation with metal oxides, polymers, and other 2D materials. A comparative analysis of the RT NO_2_ sensor performance based on MXenes and their hybrids is provided. We also discuss the major challenges of using MXene-related materials and the areas that can further advance in the future for the development of high-performance room temperature NO_2_ gas sensors.

## Abstract 2

MXenes with distinctive structures, good electrical conductivity and abundant functional groups have shown great potential in the fabrication of high performance gas sensors.

## Graphical abstract

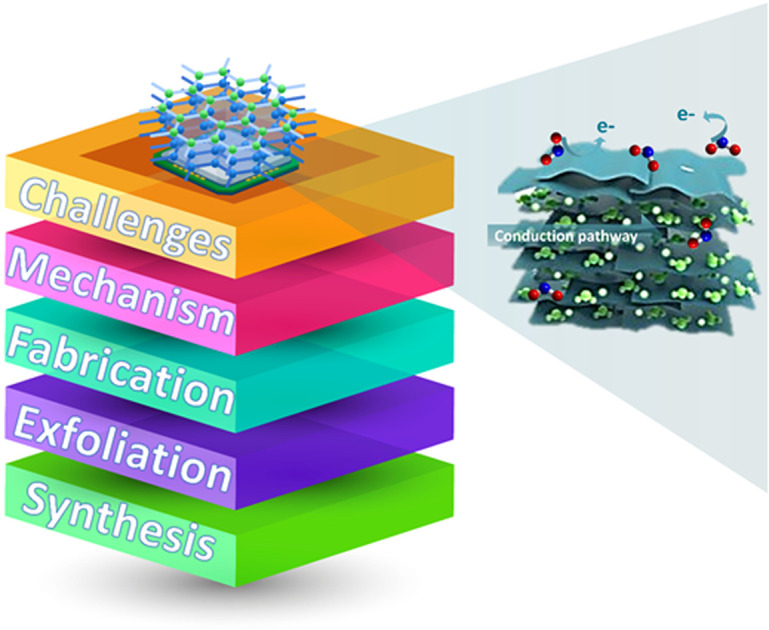

## 1. Introduction

Human health has been severely harmed as a result of air pollution caused by urbanisation. Along with medical and technological advancements, the usage of synthetic fertilisers for greater crop production has resulted in steady population growth while also increasing nitrogen dioxide (NO_2_) gas emissions into the atmosphere. NO_2_ constitutes one of the most toxic gases, with a pungent odour, and it deteriorates human health when exposed to ppm levels for an extended period of time.^1–3^ When compared to CO_2_, NO_2_ is one of the main greenhouse gases that contribute to global warming and is responsible for stratospheric ozone depletion.^4–6^ Hence, the design and fabrication of high performance NO_2_ sensors operated at room temperature are very important to monitor the presence of low concentration gas molecules effectively. Accurate measurement of NO_2_ gas under real environmental conditions at room temperature with high selectivity, and reversibility at low cost is a challenging task.^7–9^

In the past few decades, electrical signal-based NO_2_ gas detection has been reported using sensors made of a variety of materials including metallic nanoparticles/metal-oxide,^10–14^ polymers^13,15–17^ and two-dimensional materials (2D) such as MoS_2_.^18–21^ Recently, there has been a lot of interest in research on 2D materials, including MXenes, because of their properties like high surface area to volume ratio, layer-dependent tuneable mechanical, electrical, optical, and physicochemical properties arising from quantum confinement, and low dimensionality effects.^22–24^ Among these 2D materials, graphene and transition metal dichalcogenide (TMDs)-based gas sensors are widely explored due to their excellent mechanical properties, high carrier mobility, and remarkable electrical and optical properties. Despite having an excellent sensor response and response time, the NO_2_ sensors based on graphene suffered from long-recovery time whereas TMD-based sensors suffer from incomplete recovery due to its high adsorption.^25^ This limitation motivated researchers to explore other 2D materials including MXenes. The interaction of gas molecules with sensing materials is an indelible feature of any gas-sensing process. Recently, MXene-based gas sensors have received a lot of attention due to their several advantages. Further, they have already shown applications in the fields of electrochemical energy storage devices, flexible electronic devices and so on due to their excellent thermal and chemical stability. This family of 2D transition metal nitrides/carbides that possess intrinsic metallic conductivity demonstrate excellent gas sensing performance due to the properties ascribed above.^26^ MXenes have the chemical formula of M_*n*+1_X_*n*_T_*x*_ (*n* = 1–4) where M represents the transition metal, X represents C or N, and T_*x*_ corresponds to the functional groups –OH, –O, –F, *etc.*^27,28^ MXene-based materials have attracted considerable interest for applications in gas sensing due to a variety of advantages such as high surface area, metallic conductivity, high mechanical flexibility, hydrophilicity, presence of abundant active sites, tuneable surface chemistry, and improved stability, among others.^29–32^ Various approaches, such as doping, defect and vacancy engineering, heterostructure formation and modification with charge blocking layers and functional groups, and so on, are also used to improve the performance of the sensor in terms of its selectivity, limit of detection (LOD), response and recovery times, *etc.*

**Fig. 1:**
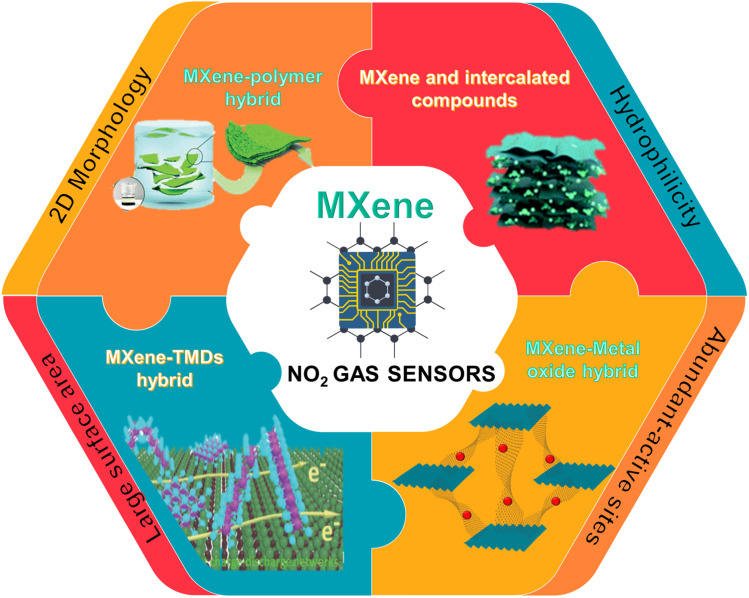
A Scheme on the summary of the content of the review and with a part describing the advantages of MXenes for gas sensing, reprinted in part permission from *Adv. Funct. Mater.* 2020, **30**, 0190302, Copyright (2023), Wiley *Materials and Adv. Funct. Mater.* 2020, **30**, 2005305, Copyright (2023), Wiley Materials.

Among the various reported MXenes, Ti_3_C_2_T_*x*_ is the most explored one for gas-sensing applications. Since the average thickness of the reported Ti_3_C_2_T_*x*_ layer (∼2 nm) is much less than the depletion layer thickness, the sensing mechanism is expected to be a surface-dominated process. As a result, this kind of MXene-based gas sensor can operate at room temperature.^33^ However, Ti_3_C_2_T_*x*_ has drawbacks such as slow response kinetics and irreversibility that limit its use in RT gas sensor technology. Literature studies proved that modification of the constituents is not the only factor affecting the properties of MXene but functional group modification also plays an important role in determining its optical, mechanical and electrical properties. Theoretical studies proved that O-terminated MXenes are the best candidate for NH_3_ sensing due to their semiconductor electronic characteristics. Given the comparable atomic structures of the MXenes with different terminations, one wonders whether these new forms of MXenes could be used as NO_2_ gas sensors.^34^ In this review, we focused on and discussed the recent literature on NO_2_ sensors operated at room temperature based on pristine and heterostructures of MXenes. We discussed the sensing mechanisms and different approaches to tune the sensing performance. Finally, the challenges and future perspectives of this research field for the development of high performance NO_2_ sensors are discussed. Fig. 1 summarizes the major contents of the review.

## 2. NO_2_ gas sensing mechanisms of 2D materials and MXene-based sensors

In this section, we will discuss the mechanism involved in the MXene-based NO_2_ gas sensors. NO_2_ is generally a secondary product primarily generated from NO sources as given by eqn (1)

1 2NO + O_2_ → 2NO_2_

Because of the unpaired electron character of nitrogen in NO_2_, it has an electron-accepting nature and acts as a strong oxidizing agent. Hence, the electrons from the sensing materials are taken by NO_2_ molecules.^35^

**Fig. 2:**
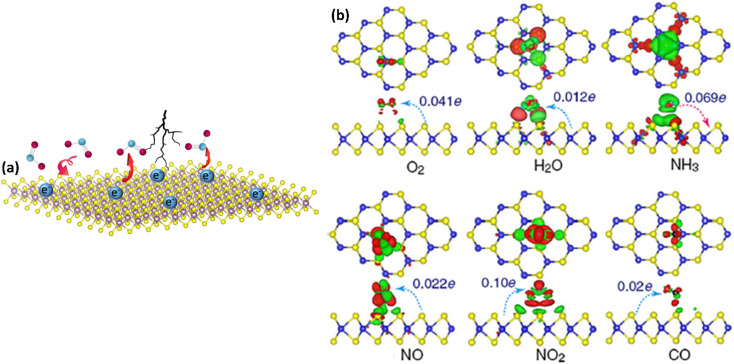
A schematic representation of the charge transfer mechanism caused by gas adsorption on layered 2D materials. (a) The transport of electrons to and from adsorbent materials while gas molecules interact with the surface, depending on the distance, site of adsorption, gas type, molecule orientation and (b) charge transfer mechanism and density difference plots for O_2_, H_2_O, NH_3_, NO, NO_2_, and CO interacting with monolayer MoS_2_, reproduced with permission from 2013 Yue *et al.*; license Springer.^38^

The sensing mechanisms of 2D material-based gas sensors are primarily explained using two well-established models. Specifically, (i) charge transfer mechanisms and (ii) the ionosorption model. In the case of charge transfer mechanisms, electrons or holes act as the charge carriers depending on the type of materials (*i.e.*, p-type or n-type) being used as the active component of the sensor device (Fig. 2). Furthermore, the direction of the flow of charge transfer depends upon whether reducing or oxidising gas molecules are used as analytes. Additionally, the reactivity of the adsorbates and adsorbents, and their adsorption energy all affect how the analytes interact with the sensing materials.^9,36,37^ The schematic representation of the charge transfer process used in 2D material-based gas sensors is shown in Fig. 2.^38^

MXenes have been investigated as an active material for gas sensing applications. As previously stated, the high conductivity, huge specific surface area, hydrophilicity and surface terminations of MXenes make them attractive for use in NO_2_ gas sensors.^39^ The fundamental sensing mechanism of pure MXenes involved adsorption or desorption of gas analytes onto the sensing layer. The majority of the presented research elucidated the process involved in reducing gas sensing; however, they did not address the interaction of oxidising gases such as NO_2_.^32^ In 2017, it was discovered that Ti_3_C_2_T_*x*_ responds positively to reducing gases (methanol, ethanol, ammonia, and acetone). As a result, it was hypothesised that Ti_3_C_2_T_*x*_ was a p-type semiconductor and the gas sensing response was caused by the predominant charge carrier transfer by the interaction between the gas analyte and Ti_3_C_2_T_*x*_.^40^ In 2018 and 2019, it was found that MXene-based gas sensors always provide a positive response for all gases, regardless of their kind (oxidising or reducing).^40–42^ Thus, p-type semiconductor technology is not the appropriate mechanism for MXene gas sensing. Therefore, two additional factors were presented to represent the positive resistance change towards various gas analytes (i) MXene is a metallic compound rather than a semiconductor and this metallic sensing layer always hinders the charge-carrier transport.^41^ This behaviour is completely distinct and is independent of the electron-donating/accepting properties of analytes and the dominant charge carrier type (*i.e.* p or n) of the sensing channel. In this context, (ii) interlayer spacing could be another cause for MXene's increased resistance to different gas analytes. Gas sensing, which occurs as a result of interlayer swelling after gas adsorption, impedes out-of-plane electron transport and increases electrical resistance.^43^ The MXenes' metallic conductivity and interlayer spacing is a one-of-a-kind trait that occasionally makes the gas sensing process somewhat different and more exciting than the sensing mechanism of normal semiconducting materials. The surface functional groups also play a major role in the sensing mechanism as the hydrophilic group (–OH and =O) enhances the gaseous interaction with MXenes. Besides functional groups, terminal groups also affect the sensing mechanism. For example, O-terminated MXenes are the best candidates for NH_3_ sensing due to their semiconductor electronic characteristics.^32^ In other studies, done by Hu *et al.* they demonstrated that S-terminated MXene is the best candidate for NO_*x*_ gas sensors.^34^

Besides the charge-transfer mechanism, Zhang *et al.* employed an ionosorption model to better understand the sensing mechanisms of V_2_CT_*x*_ MXenes, which could also be applied to other MXenes and hybrids.^44^ Here O_2_^−^_(ads)_ ions interact and contribute to sensing processes at room temperature or low temperatures. Because this review is about MXene-based room temperature sensors, O_2_^−^_(ads)_ oxygen ion species play an important role here, and other oxygen species contribute to gas sensors that work at higher temperatures.^45–47^ O_2_^−^_(ads)_ oxygen ions are adsorbed over the surface of active sensor materials such as MXenes at ambient temperature *via* the following reactions:

2 O_2(gas)_ → O_2(ads)_

3 O_2(ads)_ + e^−^ → O_2_^−^_(ads)_ (room temperature)

**Fig. 3:**
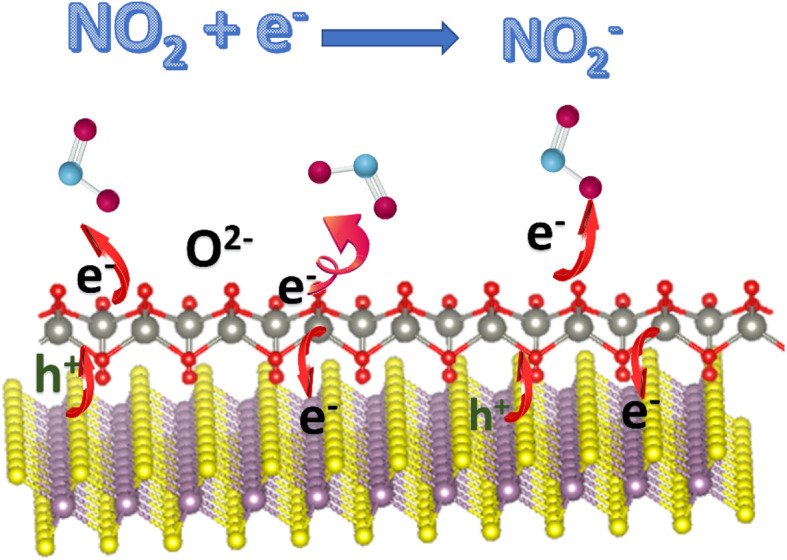
NO_2_ sensing mechanism based on an ionosorption model for 2D materials.

The width of the junction potential and the electron depletion layer at the grain boundaries increase when the gas sensors come in contact with an oxidising gas like NO_2_. This is because the gas molecules not only absorb electrons from the active materials but also interact with the adsorbed oxygen ion species (O_2_^−^_(ads)_). Following are several formulas for the chemical reaction caused by the interaction of NO_2_ gas molecules at room temperature or at low temperatures (Fig. 3):

4 NO_2(gas)_ + e^−^ → NO_2_^−^_(ads)_

5 2NO_2_^−^_(ads)_ + O_2_^−^_(ads)_ + e^−^ → 2O^−^_(ads)_ + 2NO_2_^−^_(ads)_

When air is introduced to gas sensors in the reverse process, NO_2_^−^ interacts with the holes and releases electrons once more, resulting in the formation of NO_2_ gas molecules.

6 NO_2_^−^_(ads)_ + h^+^ → NO_2(g)_

The above-given reactions are the essence of MXene-based RT NO_2_ sensors. Fig. 3 describes the schematic illustration of the NO_2_ sensing mechanisms based on the ionosorption model.^48^

Also, the synergistic and reverse enhancement effect in MXene composites directly influences the NO_2_ sensing mechanism depending on the type and properties of the foreign materials. Generally, MXenes are reported as a channel layer and metal oxides are used as a supporting layer during composite formation. In this case, the basic mechanism is very similar to that of the conventional metal oxide semiconductor-based sensors in which effective absorption/desorption of the gas analyte on the surface of sensing materials shows an effective change in device resistance. The gas sensing mechanism of MXene-metal oxide composites is connected to the interfacial interactions and heterojunction development of both involved materials. The type of response (p- or n-type) following composite formation is entirely dependent on the composition of both materials as well as the material that plays the predominant role in carrier conduction.^35^ Gasso *et al.* synthesized a WO_3_/Ti_3_C_2_T_*x*_ hybrid for NO_2_ sensing where this composite shows electron transfer from MXene to WO_3_ leading to the formation of heterojunctions. The oxygen adsorption on the surface leads to an electron depletion layer (EDL) and hole accumulation layer (HAL). When the sensor is exposed to NO_2_ it captures electrons from the conduction band of WO_3_ that lead to an increase in resistance.^49^

The gas sensing mechanism of oxidised MXenes is generally considered as a Schottky barrier (SB) modulation. The oxidizing tendency of Ti_3_C_2_ flakes to form a heterojunction consisting of metallic Ti_3_C_2_ and semiconducting TiO_2_ is utilized to form the SB sites. Here Ti_3_C_2_ MXene with intrinsic metallic conductivity offers the possibility of SB modulation within the sensing channel itself. Choi *et al.* demonstrated this *in situ* formation of multiple SBs in a single NO_2_ gas sensing channel. In the presence of oxidising gas such as NO_2_ SB can be shifted upwards which surpasses the transport of electrons and lead to a high gas-sensing response. The sensing mechanism for MXene-polymers also depends upon a number of factors including redox reactions between the gas analytes and hybrids, and charge carrier concentration changes happening in sensing layers.^50^ Zhao *et al.* reported the NO_2_ sensing mechanism of a poly(l-glutamic acid) (γ-PGA)-MXene sensor which is different from the conventional metal oxide semiconductor-based sensors. Here, the gas molecules are adsorbed on the γ-PGA using a non-covalent bond. In the presence of NO_2_, the resistance of the sensor changes from a negative response to a positive response. Water molecules in the presence of air adsorbed onto the γ-PGA film and hydrolysed. At high concentrations of NO_2_ excess molecules are adsorbed onto the surface *via* hydrogen bonding and electrostatic interaction and it may compete with H_2_O molecules for adsorption by the equation given below; this hinders ion conduction, thereby increasing the resistance. Here, the blocking behaviour of MXene was enhanced by γ-PGA and the acceleration rate was also increased.^51^

7 NO_2_ + H_3_O^+^ + e^−^ ↔ NO + 2H_2_O

Aside from the synthesis method and etching conditions, the MXene surface termination has a considerable impact on NO_2_ gas sensing. For example, it was recently found that NaOH alkalization can shift the response of the Ti_3_C_2_T_*x*_ type to negative.^52^ According to XPS measurements, alkalization increased the O/F ratio of the termination on this Ti_3_C_2_T_*x*_ MXene from 2.6 to 7.6. As a result, it was claimed that a high O/F termination ratio can change the response type from positive to negative. However, the underlying reasons behind this conversion remain unknown, and it is not clear whether this conversion occurs in other MXenes.^52^ According to Zhang *et al.*'s studies, this alkalization treatment with DMSO improves the NO_2_ gas sensing performance of V_2_CT_*x*_ MXene, indicating that this conversion also occurs with other MXenes.^44^ They demonstrated that the adsorbed H_2_O and O results in p-type sensing behaviour in V_2_CT_*x*_. With the exposure of V_2_CT_*x*_ MXene-based sensors to humid air, the oxygen molecules absorbed on the surface of V_2_CT_*x*_ ionized to O_2_^−^ with the consumption of electrons. As a result, the concentration of the V_2_CT_*x*_ MXene major charge carrier increases, increasing conductivity and decreasing resistance. When NO_2_ molecules are exposed to the V_2_CT_*x*_ MXene-based sensors, they are adsorbed on the active sites by surface terminations such as –OH and –O. Electrons can be transferred from the V_2_CT_*x*_ to NO_2_ gas molecules. This charge transfer results in increasing the hole concentration of MXene, which further lowers the resistance and the conductivity of V_2_CT_*x*_ MXene-based sensors increase. The studies carried out by Choi *et al.* also prove that the response of Mo_2_CT_*x*_ MXene using tetramethylammonium hydroxide (TMAOH) as the intercalant can change the response type which can be attributed to the high density of MXene surface functional groups and its intrinsic metallic conductivity.^53^ The DFT studies demonstrated that TMA intercalated MXenes show high adsorption energy towards NO_2_

## 3. Recent advancements in NO_2_ gas sensors using MXene-based materials

MXenes are an excellent choice for gas-sensing applications due to their abundant active sites, large aspect ratio, availability of abundant functional groups, hydrophilicity, metallic conductivity and tunable surface chemistry. However, MXenes show disadvantages such as irreversibility and low response kinetics, which limit their gas sensing applications.^51^ The development of MXene heterostructures to modify the physiochemical properties of MXenes is a possible route for the development of high-performance low-temperature sensors. Surface modification, functionalization with noble metals, additive doping, inorganic heterojunction sensitization, light activation, *etc.* are some of the other approaches which can further tune the properties of MXenes and make them better candidates for RT NO_2_ gas sensing. MXenes have a massive gas adsorption area, variable layer numbers, and an interlayer swelling effect, making them an amazing gas-sensitive material with a good signal-to-noise ratio.^44,54–56^Table 1 summarizes the reported NO_2_ sensing performance of different MXenes and their hybrid-based sensors.

**Table 1:** Comparison table showing the performance of MXene-based NO_2_ gas sensors at room temperature

Materials	Dynamic range	Sensor performance, *S* = *R*_a_/*R*_g_, *t*_res_ = response time, *t*_rec_ = recovering time, LOD = limit of definition	Sensing environment RT = room temperature (temp., dry air, RT, *etc.*)	Reference
Mo_2_CT_*x*_	0.125–5 ppm	*S* = 18.2	RT, N_2_	53
Nb_2_CT_*x*_	5–25 ppm	LOD = 67 ppb	RT, air	56
		*t*_res_ = 39 s		
		*t*_rec_ = 66 s		
		*S* = 16.01 for 25 ppm		
Nb_2_CT_*x*_-CTAB	5–25 ppm	LOD = 21 ppb	RT, air	56
		*t*_res_ = 39 s		
		*t*_rec_ = 78.9 s		
		*S* = 50.23 for 25 ppm		
V_2_CT_*x*_	5–50 ppm	*t*_res_ = 76 s	25 °C/RT, air	60
		*t*_rec_ = 20 s		
		*S* = 70 for 50 ppm		
SnO_2_-MXene	1–960 ppb	*S* = 23% for 30 ppb	RT, air	68
		*t*_res_ = 146 s		
		*t*_rec_ = 102 s		
Microwave irradiated SnO_2_-MXene	0.1–10 ppm	*S* = 24.8 for 10 ppm	150 °C, air	67
		LOD = 0.0153 ppm		
Facet controlled SnO_2_/Ti_3_C_2_	0.05–10 ppm	Response = (Δ*R*/*R*_a_) = 0.02–1.57	25 °C/RT, air	66
Self-powered SnO_2_ fibre/sodium l-ascorbate-treated MXene	—	*t*_res_ = 265 ms	RT, air	69
		*t*_rec_ = 75.5 ms		
		LOD = 0.03 ppb NO_2_		
Ti_3_C_2_/TiO_2_	0.125–5 ppm	*S* = 16% for 5 ppm	RT, air	53
		LOD = 125 ppb		
ZnO-Ti_3_C_2_T_*x*_ UV excited	5–200 ppb	*S* = 81% for 50 ppm	RT, air	70
		LOD = 0.2 ppb		
		*t*_res_ = 17 s		
		*t*_rec_ = 24 s		
Ti_3_C_2_T_*x*_-ZnO sphere	5–100 ppm	*S* = 41.93% for 100 ppm	RT, air	71
		*t*_res_ = 53 s		
		*t*_rec_ = 103 s		
Ti_3_C_2_T_*x*_-ZnO nanosheets	—	*S* = 367.63% to 20 ppm	RT, air	73
		*t*_res_ = 22 s		
		*t*_rec_ = 10 s		
Ti_3_C_2_T_*x*_-CuO	1–50 ppm	*S* = 56.9% for 50 ppm	RT, air	74
		*t*_res_ = 16.6 s		
		*t*_rec_ = 31.3 s		
Ti_3_C_2_T_*x*_/TiO_2_/rGO	10–500 ppb	19.85% for 5 ppm	RT, air	26
WO_3_/Ti_3_C_2_T_*x*_	15–500 ppb	*t*_res_ = 182 s	RT, air	49
		*t*_rec_ = 75 s		
(Self-powered) Ti_3_C_2_T_*x*_/WO_3_	0.5–50 ppm	*S* = 510% for 50 ppm	RT, air	75
Ti_3_C_2_-WO_3_	30–1000 ppb	*S* = 19% for 30 ppb	RT, air	76
		*t*_res_ = 25 s		
		*t*_rec_ = 40 s		
BiOCl-MXene	100 ppm	*S* = 34.58	RT	77
		*t*_res_ = 3.15 s		
		*t*_rec_ = 31.05 s		
MXene-PEODT/PSS	1 ppm	—	65 °C	91
CO_3_O_4_@PEI/Ti_3_C_2_T_*x*_	0.3–100 ppm	*S* = 27.9% @ 100	RT, air	78
α-MOC_1−*x*_	0.125–5 ppm	*S* = 15	RT, air	85
		LOD = ppb–ppt level		
Ti_3_C_2_T_*x*_/WS_2_	0.1–20 ppm	*S* = 15.2% @ 1 ppm	RT, air	86
		LOD = 11 ppb		
		*t*_rec_ = 60 s		
2H MoS_2_/TiC_3_T_2_T_*x*_	30–70 ppm	65.6% @ 100 ppm	RT, air	18
MoS_2_/Ti_3_C_2_T_*x*_	1–50 ppm	40% @ 20 ppm	RT, air	19
		*t*_res_ = 525 s		
		*t*_rec_ = 155 s		
Mo_2_TiC_2_T_*x*_/MoS_2_	0.2–50 ppm	*S* = 415.8% @ 50 ppm	RT, air	21
		LOD = 2.5 ppb		
		*t*_res_ = 33.5 s		
		*t*_rec_ = 140.1 s		
Black phosphorous QDS/Ti_3_C_2_T_*x*_	50 ppb–10 ppm	LOD = 13 ppb	RT, air	90
		*t*_res_ = 72 s		
		*t*_rec_ = 85 s		
Nb_2_CT_*x*_-APTES	5–25 ppm	*S* = 31.52% @ 25 ppm	RT, air	65
		LOD = 15 ppb		
		LOQ = 52 ppb		
		*t*_res_ = 36 s		
		*t*_rec_ = 96.8 s		
Ti_3_C_2_T_*x*_/γ-PGA	2–50 ppm	*S* = 1127.3%	RT, air	96
		*t*_res_ = 43.4 s		
		*t*_rec_ = 3 s		
Ti_3_C_2_/TiO_2_	1–100 ppm	*S* = 19.76 for 100 ppm	RT, air	98
Ti_3_C_2_T_*x*_@TiO_2_@MoS_2_	0.02–50 ppm	*S* = 55.16 (*R*_a_/*R*_g_) @ 50 ppm	RT, air	98
		*t*_res_ = 1.8 s		
		*t*_rec_ = 50 s		
		LOD = 23 ppb		
MXene-derived TiO_2_-rGO	0.05–20 ppm	*S* = 400% for 20 ppm	RT, air	99
		LOD = 50 ppb		
		*t*_res_ = 130 s		
		*t*_rec_ = 230 s		
MXene-derived TiO_2_-SnS_2_	—	115 for 1000 ppm NO_2_	RT, air	100

### 3.1. Pristine and intercalated MXenes

The theoretical studies conducted by Yu *et al.* first revealed the possibilities of MXenes for gas sensing applications. Their DFT analyses demonstrate that other gas molecules, notably NO_2_, display distinct adsorption behaviours compared to NH_3_. Lower adsorption energies of NO_2_ gas molecules on the Ti_2_CO_2_ monolayer reflect weaker interactions between MXene and NO_2_, implying that this Ti_2_CO_2_ MXene is unsuitable for NO_2_ gas sensing applications.^57^ Furthermore, Jian *et al.* revealed that the Ti_3_C_2_T_*x*_ MXene-based gas sensor is not suited for strong and moderate oxidising gases such as NO_2_ due to its high proclivity to oxidise to TiO_*x*_.^33^

The other pristine MXenes which are reported to show promising NO_2_ sensing performance are Mo_2_CT_*x*_, Nb_2_CT_*x*_, and V_2_CT_*x*_.^44,53,56,58,59^ For example, Molybdenum carbide-based NO_2_ sensors demonstrated a high signal-to-noise ratio with the ability to detect NO_2_ concentration even at the ppb level and high ambient stability due to their high electrical conductivity, rich density of states near the Fermi level, superior catalytic properties, good resistance to corrosion and low chemical reactivity.^59^ According to reports, Mo_2_CT_*x*_ MXene exhibits a three-phase transition to gas sensing behaviour. Here, the film thickness and the presence of organic intercalants play a crucial role in tuning the performance.^53^ It is demonstrated that the thin film sensor with 5 nm thick Mo_2_CT_*x*_ intercalated by tetramethylammonium hydroxide (TMAOH) shows a p-type gas sensing response whereas it displayed an n-type response without intercalants and films with thickness above 700 nm exhibit conductor type response. Because of the stronger gas molecule binding in the presence of the intercalants, the intercalation technique allows the gas molecules to penetrate, resulting in increased gas sensing performance with sensitivity 30 times greater than that of the deintercalated films. The effect of the thickness of the film is also studied and it demonstrates that the thin film of Mo_2_CT_*x*_ shows a better response towards NO_2_. This is because as the thickness of the film reduces, gas molecules can more easily penetrate into the interlayer space, adsorbing on the surface of MXene flakes faster and resulting in a quick response and short recovery time.^53^

Rathi *et al.* employed Nb_2_CT_*x*_ to demonstrate that the strong metallic conductivity of MXenes is beneficial for noise reduction and that the abundance of functional groups is favourable for achieving higher sensitivity.^56^ It has been found that the sensor response of Nb_2_CT_*x*_ MXenes treated with cetyltrimethylammonium bromide (CTAB) increases thrice when compared to pure MXenes. The activation of the exposed surface, as well as the interlayer swelling with the hydroxyl groups, play an important role in the adsorption of target molecules, resulting in increased sensing characteristics for delaminated MXene by CTAB.

V_2_CT_*x*_ MXenes intercalated by Na^+^ ions also show 80 times higher response than the as-prepared samples for NO_2_ gas (5–50 ppm).^60^ Intercalation by Na atoms swelled the layers of the MXenes which allowed the analyte gases to enter inside for better interaction. Further, the existence of the surface functional groups (–OH) allowed the adsorption of water molecules on MXenes leading to more reactions with NO_2_ to form NO (3NO_2_ + H_2_O → 2HNO_3_ + NO). Hence, the swelling effect and surface adsorption promoted by the functional groups present in MXenes contributed significantly to achieve enhanced NO_2_ sensing performance.^60^

#### 3.1.1. MXene modification

Studies revealed that MXenes can be stored for a long time at low temperature, whereas their stability is very low in aqueous solution at RT or high temperature. Thus, to investigate the electrical characteristics and environmental stability of Ti_3_C_2_T_*x*_, Lipatov and colleagues fabricated field-effect transistors (FETs) with single-layered Ti_3_C_2_T_*x*_ flakes as the conductive channels. The results showed that single-layered Ti_3_C_2_T_*x*_ flakes had more field-effect electron mobility and resistivity than bulk Ti_3_C_2_T_*x*_. According to the environmental stability data, these FETs based on Ti_3_C_2_T_*x*_ remain stable and highly conductive even after 70 hours of exposure to humid air.^61^ Even though MXenes have great sensitivity and detection limits, their stability in oxidative environments, which is rarely discussed in the literature, is a key limitation in the fabrication of RT-based NO_2_ sensors. MXenes will rapidly degrade over time in the presence of air or in the presence of water and their hydrophilic properties make them more vulnerable to oxidation.^35^ For example, Ti_3_C_2_T_*x*_ undergoes oxidation while annealing at high temperatures and also under plasma treatment and results in the formation of TiO_2_ which possesses higher oxygen adsorption capacity and electronic transmission.^62,63^ The functional groups also strongly affect the electronic properties as well as the work function of MXenes. With the help of inverse photoelectron spectroscopy, May *et al.* studied the work function variation of MXenes with the functional groups. Their research demonstrated that heating MXenes in a vacuum atmosphere raises their work function from 3.9 to 4.8 due to water adsorption, –OH species, and carbon-dominated contaminations.^35,63^ These studies prove that MXene stability is very important during the fabrication of gas sensors.

Compared to other MXenes Ti_3_C_2_T_*x*_ is not much preferred for oxidising gases such as NO_2,_ because it gets easily oxidised to TiO_2_. The studies done by Jian *et al.* showed that Ti_3_C_2_T_*x*_-based gas sensors give stable response–recovery curves for both reducing and oxidizing gases where this sensor shows a major baseline resistance drift in the case of NO_2_ gas sensing. This irreversible performance towards NO_2_ gas could be attributed to Ti_3_C_2_T_*x*_ oxidation in an oxidising environment. Hence, compared to other MXenes Ti_3_C_2_T_*x*_ is not much preferred for oxidising gases such as NO_2,_ because it gets easily oxidised to TiO_2_.^33^ In this scenario, Chae *et al.* stored Ti_3_C_2_T_*x*_ in an aqueous solution at different temperatures and aged for different time intervals. Their studies concluded that MXene which is stored at −80 °C for 5 weeks and as-synthesized samples show the same response towards NO_2_ at 5 ppm demonstrating that the oxidation stability can be controlled by maintaining the storage conditions.^64^ To address these issues of oxidation, several techniques including modification with a hydrophobic flurorosilane layer, and polymers were done. This surface modification helps to decrease MXene oxidation and also allows the simultaneous introduction of additional reactive groups. For example, Naveen Kumar *et al.* demonstrated that the introduction of amine groups over the Nb_2_CT_*x*_ MXene helps in the detection of acidic gases such as NO_2_ by acting as an electron acceptor.^65^ Rathi *et al.* also modified Nb_2_CT_*x*_ MXene with CTAB and here the stability enhancement was ascribed to the (i) non-covalent bonding between CTAB and Nb_2_CT_*x*_ MXene (ii) the long hydrophobic chains in CTAB hinders the interaction of humidity and oxygen with Nb_2_CT_*x*_ MXene. CTAB functionalization can aid in the bulk manufacture of stable MXenes under ambient conditions for future device manufacturing in NO_2_ gas sensors.^56^

### 3.2. MXene-based hybrid materials

#### 3.2.1. MXene-metal oxide hybrids

**Fig. 4:**
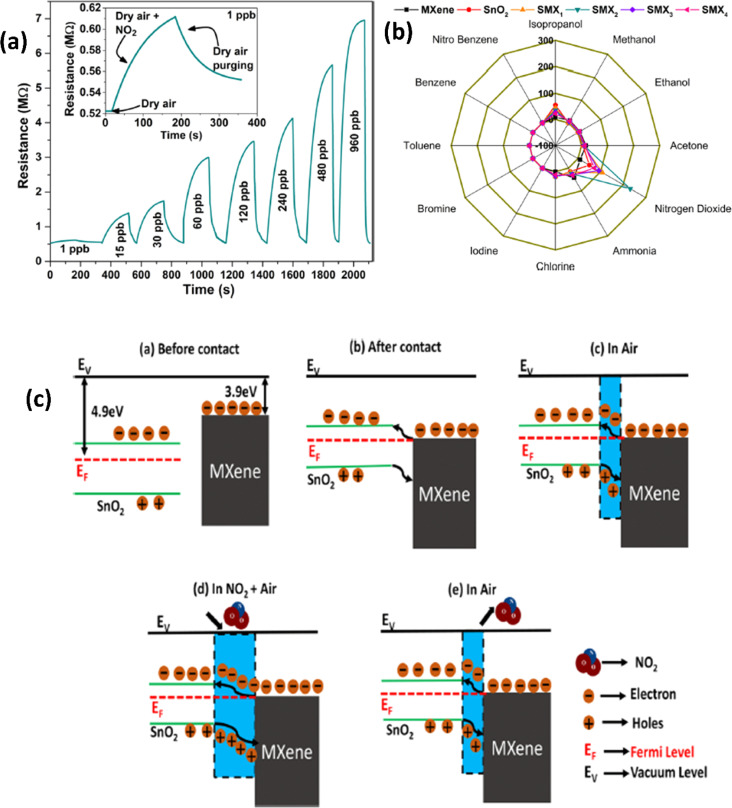
(a) Resistance–time curves for the SnO_2_/MXene sensor with exposure to different concentrations of NO_2_. (b) Polar plots for various gases at 30 ppb concentrations for pristine SnO_2_, MXenes and their heterostructures at room temperature. (c) Schematic representation of the NO_2_ sensing mechanism of the SnO_2_/MXene heterostructure, reprinted in part permission from ref. 60 Copyright (2023) Elsevier.

To avoid restacking of MXene sheets and to retain the high surface area, hybrid materials with metal oxides have been explored to achieve improved NO_2_ sensing properties.^66–68^ Due to the presence of abundant surface adsorption species and high gas sensing capabilities, metal oxides such as CuO, TiO_2_, WO_3_, BiOCl, Co_3_O_4_, *etc.* have been used to fabricate hybrid materials with MXenes, and they displayed promising NO_2_ sensing properties. Gasso *et al.* revealed that by regulating the SnO_2_ loading in the MXene-SnO_2_ heterostructures, high sensor performance in terms of selectivity, sensitivity, reproducibility, and repeatability can be achieved under humid conditions.^68^ Sensors based on 20 wt% SnO_2_ in MXene-SnO_2_ heterostructures had a nearly 5 times greater response (231%) to 30 ppb NO_2_ at ambient temperature, with a quicker recovery time (146 s) and response time (102 s) than pure SnO_2_ (Fig. 4). In another report, it is shown that the microwave-irradiated SnO_2_ (2 wt%)-Ti_3_C_2_ nanocomposites exhibited a superior response of 24.8 for 10 ppm NO_2_ as compared to the pristine MXene, SnO_2_ and un-irradiated SnO_2_-MXene nanocomposite-based gas sensors.^67^ Liu *et al.* reported that by regulating the co-exposed (110) and (221) SnO_2_ facets in the MXene-SnO_2_ nanocomposites, excellent sensing properties in terms of linear response (*R*^2^ = 0.99729), good selectivity and recycling performance can be achieved.^66^ NO_2_ sensing mechanisms of SnO_2_-MXene hybrids involve a charge transfer process where the adsorbed oxygen ion species played an important role as per eqn (2) and (3) (Fig. 4c). These adsorbed oxygen ions trap electrons from the SnO_2_ conduction band, forming the electron depletion layer (EDL). Due to their mismatched work function (SnO_2_ = 4.9 eV and MXene = 3.9 eV) and Fermi level locations, metallic MXene and semiconducting SnO_2_ heterojunctions generate Schottky barriers. To reach equilibrium, electrons from MXene move to SnO_2_ until equilibrium is reached, resulting in band bending. When exposed to air, the adsorbed oxygen species (O_2_^−^) form the EDL and hole accumulation layer (HAL) at the heterojunction interface, and these layers interact with the NO_2_ gas as shown in eqn (4) and (5). Finally, NO_2_ molecules take electrons from the heterostructure's surface, increasing the EDL width and trapping a high number of holes in the HAL, thus lowering the total conductivity. Gasso *et al.* recently developed a self-powered humidity-tolerant gas sensor made up of MXene treated with sodium l-ascorbate and SnO_2_ nanofibers. This SnO_2_/MXene nanocomposite shows excellent response towards NO_2_ when compared to pristine MXene and SnO_2_ with a limit of detection around 0.3 ppb.^69^

**Fig. 5:**
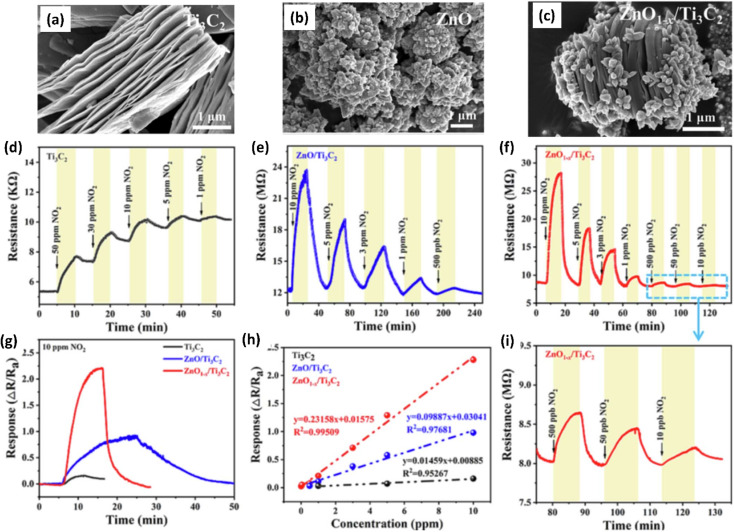
SEM images showing morphology of (a) pristine Ti_3_C_2_ (MXene), (b) ZnO and (c) ZnO/Ti_3_C_2_ (MXene), (d–f) Response/recovery curves of ZnO/Ti_3_C_2_ (MXene) (g) response curves and (h) linear fitting curves of Ti_3_C_2_ (MXene) and ZnO/Ti_3_C_2_ (MXene) heterostructures at room temperature to gas NO_2_. (i) Response/recovery curves of ZnO/Ti_3_C_2_ (MXene) heterostructures at different concentrations of NO_2_ (10, 50 and 500 ppb), reprinted with reprinted in part permission from ref. 63 from Copyright (2023) Elsevier.

Due to their high surface area, sizable active sites, and modification of the carrier density, MXene-ZnO composites are another type of MXene-metal oxide composite that have been reported to exhibit promising NO_2_ sensing capabilities.^14,70–72^ ZnO/Ti_3_C_2_T_*x*_ MXene nanocomposites displayed an improved response of 3.4 for NO_2_ (8 ppm) with a recovery time of 254 s and response times of 191 s respectively.^14^ The crumpled spheres of the heterostructures demonstrated improved NO_2_ sensing performance due to their increased surface area, an abundance of edges and flaws generated by folding, and the development of the MXene/ZnO p–n junction.^71^ This heterostructure showed enhanced response from 27.3% to 41.9% for 100 ppm NO_2_, as well as a significant increase in the recovery rate from 30% to 100%. ZnO_1−*x*_/Ti_3_C_2_T_*x*_ MXene composites with abundant oxygen vacancies showed 2.3 and 14.2-times higher response as compared to the ZnO/Ti_3_C_2_T_*x*_ and pristine Ti_3_C_2_T_*x*_ respectively with good linear response (*R*^2^ = 0.99509), long-term stability and reproducibility^14^ (Fig. 5). The sensing mechanisms for the ZnO-MXene heterostructure are predicted to follow a similar mechanism to that of the SnO_2_-MXene, in which energy bands bend and a Schottky barrier forms at the interface of the two materials.

Room temperature recovery is a big challenge for MXene-based NO_2_ gas sensors. Thus, pristine MXene-based gas sensors experience incomplete recovery at room temperature which demands sensor operation at a higher temperature. However, thermal treatment for obtaining full recovery is not suitable. Recently, light-assisted recovery of gas sensors has opened up a new prospective avenue for developing RT gas sensors. Light illumination not only aids in sensor recovery but also improves 3S performance (recovery time, low response and sensor response).^25^ Based on these, Wang *et al.* showed that under UV illumination, the NO_2_ sensing efficiency of MXene/ZnO nanorods can be considerably improved due to photo-generated electrons in ZnO reducing the depletion layers and enhancing the conduction path. The sensing response ranged from 21% to 346% for 5–200 ppb NO_2_ at room temperature, with response and recovery times of 17 s and 24 s for 50 ppb NO_2_ respectively.^70^ In the regime of portable, flexible and wearable gas sensors self-powered sensors have garnered a lot of attention. Similarly, Fan *et al.* demonstrated that MXene/ZnO nanaosheet-based sensors show enhanced performance in the presence of UV illumination. They found out that the main adsorption site for NO_2_ was present on the surface of ZnO nanosheets, while the Ti_3_C_2_T_*x*_ MXene plays a major role as a conductive path which helps to accelerate the charge carrier transformation.^73^

**Fig. 6:**
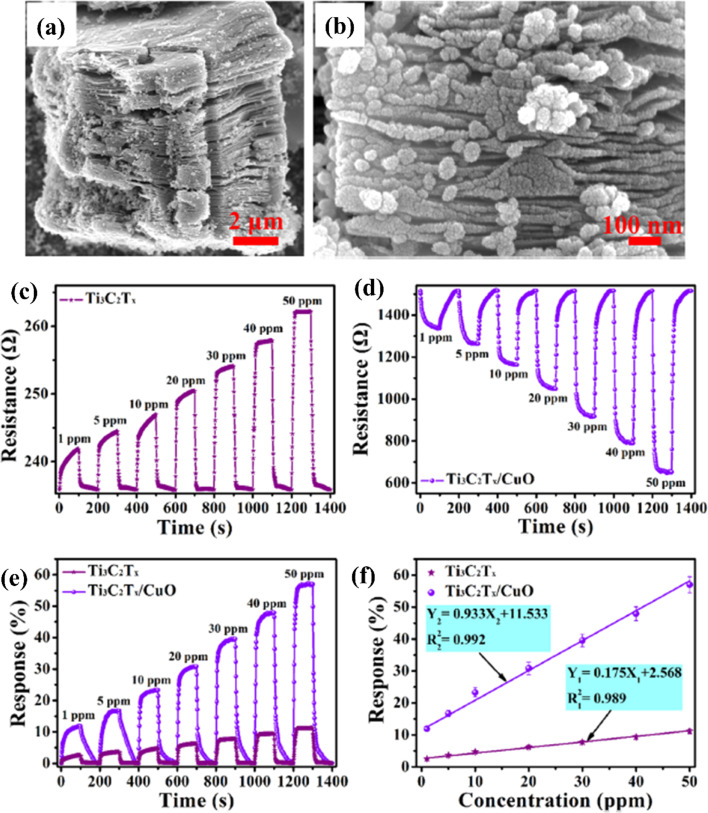
Ti_3_C_2_T_*x*_ (MXene)/CuO heterostructure for NO_2_ gas-sensing applications: (a and b) low- and high-resolution FESEM images of the Ti_3_C_2_T_*x*_ (MXene)/CuO heterostructure, (c and d) dynamic resistance curves of Ti_3_C_2_T_*x*_ (MXene) and Ti_3_C_2_T_*x*_ (MXene)/CuO heterostructure-based sensors when exposed to NO_2_ with different concentrations varying from 1 to 50 ppm at 23 °C, (e) normalized response curves of Ti_3_C_2_T_*x*_ (MXene) and Ti_3_C_2_T_*x*_ (MXene)/CuO heterostructure-based sensors, (f) normalised response curves of Ti_3_C_2_T_*x*_ (MXene) and Ti_3_C_2_T_*x*_ (MXene)/CuO heterostructure-based gas sensors at 23 °C as a function of NO_2_ gas concentration, reprinted with reprinted in part permission from ref. 67 from Copyright (2023) Elsevier.

Because of the p-type conductivity (∼1.5 eV) and narrow band gap, CuO is employed to fabricate p–n heterojunctions of CuO/MXene hybrids for NO_2_ sensing applications.^74^ (Fig. 6). For 50 ppm NO_2_, the response of mesoporous MXene-CuO nanocomposites was 5 times higher (56.99%) than pure MXene (11.7%), with ultra-fast response (16.6 s) and recovery time (31.3 s) to 20 ppm NO_2_ and notable reversibility (over 40 days).

**Fig. 7:**
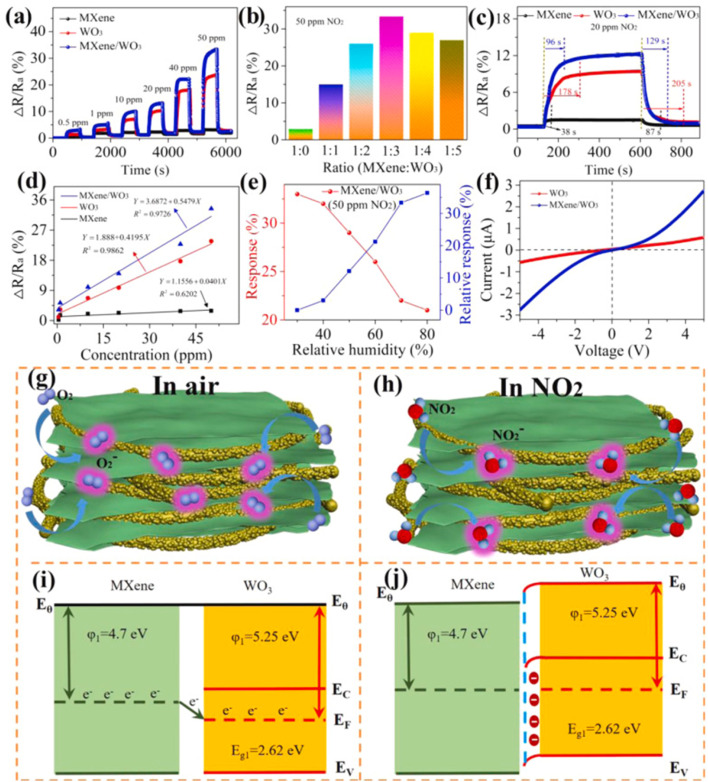
MXene/WO_3_ heterostructure for NO_2_ gas sensing: (a) the dynamic response variation of the pristine MXene, WO_3_ and MXene/WO_3_ heterostructure-based sensors at different NO_2_ concentrations. (b) The sensor response of composite materials with different mass ratios. (c) The response and recovery time of the MXene/WO_3_ heterostructure-based sensor. (d) Response–concentration fitting curves of the three developed sensors. (e) The humidity effect on the MXene/WO_3_ sensor. (f) The *I*–*V* curves of WO_3_ and the MXene/WO_3_ sensor. Schematic of the gas-sensing mechanism and energy band structure of the MXene/WO_3_ heterostructure (g and i) in the presence of air and (h and j) in the presence of NO_2_ gas, reprinted with reprinted in part permission from ref. 69 from Copyright (2023) Elsevier.

Since, theoretical calculations predicted that both Ti_3_C_2_T_*x*_ MXene and WO_3_ have significant NO_2_ reactivity with adsorption energies (*E*_ads_) of −1.12 and 0.54 eV respectively, researchers have investigated NO_2_ sensing performance of WO_3_/Ti_3_C_2_T_*x*_ MXene.^49,75,76^ Further, MXene sheets treated with sodium l-ascorbate in WO_3_/Ti_3_C_2_T_*x*_ MXene hybrids are reported to show enhanced reversibility and stability under varying humid conditions (0–99% RH).^49^ The sensing mechanisms of MXene-WO_3_ hybrids are reported to be similar to the proposed mechanisms for other metal oxide-MXene composites. For the MXene-WO_3_ heterostructures, the MXene platform for WO_3_ nanorods significantly restricted the aggregation of WO_3_ and helped to achieve increased interfacial contacts, enhanced surface area for the adsorption of gas and quicker charge transit. Wang *et al.* reported a high performing self-powered Ti_3_C_2_T_*x*_ MXene/WO_3_ sensor powered by triboelectric nanogenerators (TENGs) with a response of 510% for 50 ppm NO_2_, which was 15 times greater than that of a resistive MXene/WO_3_ sensor^75^ (Fig. 7). Heterostructures based on the highly conductive Ti_3_C_2_T_*x*_ MXene and p-type semiconductor BiOCl offered electronic transmission channels with excellent response and quick response/recovery periods, as well as a lower detection limit (50 ppb) for NO_2_ gas.^77^ Sun *et al.* constructed a NO_2_ sensor based on Co_3_O_4_ nanocrystals decorated on the surface of polyethylenimine (PEI) sheet functionalized MXene.^78^ MXene provided the electron transport channels whereas high surface area and active sites of the p-type Co_3_O_4_ nanocrystals contributed to achieving high NO_2_ sensing performance with good response and recovery times of 27.9 s and 2 s for NO_*x*_ gas at RT and at 26% RH, excellent selectivity, reproducibility and an LOD of 30 ppb. Ti_3_C_2_T_*x*_/TiO_2_/rGO heterostructure-based sensors have also been reported to have improved gas sensing performance with superior linearity, great limit of detection as low as 10 ppb, high selectivity, and response of 19.85% for 5 ppm NO_2_ at ambient temperature.^26^

#### 3.2.2. MXene-TMD hybrids

**Fig. 8:**
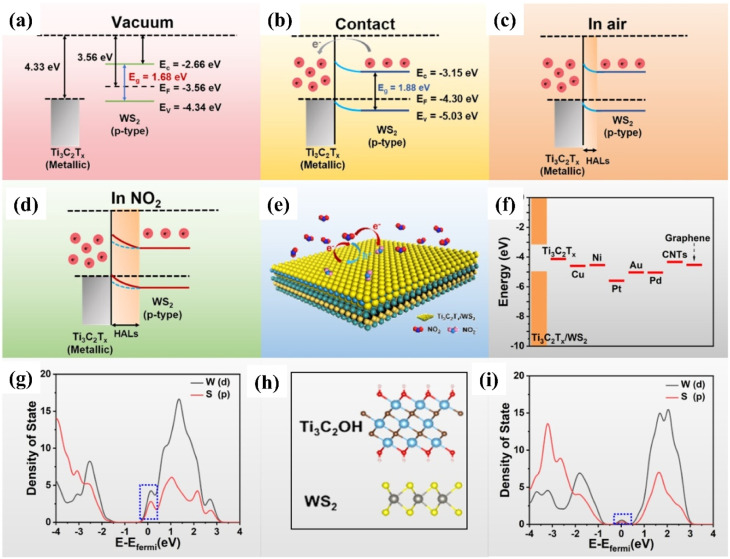
Schematic representation showing the Fermi level position and work function for theTi_3_C_2_T_*x*_ (MXene)/and WS_2_ (p-type) semiconductor (a) before contact (b) after contact (c) with air, and (d) NO_2_ at room temperature. (e) Schematic diagram of the charge transfer process of Ti_3_C_2_T_*x*_ (MXene)/WS_2_ heterostructures in the presence of NO_2_ gas at room temperature. (f) Different material work function. PDOS of the WS_2_ in contact with the (g) Au and (h) Ti_3_C_2_OH, respectively, reprinted with reprinted in part permission from ref. 80 from Copyright (2023) American Chemical Society.

Due to the sharp conductivity change upon exposure to gas molecules, 2D/2D heterostructure materials are ideal candidates for gas sensors due to improved carrier transportation, synergistic effects, combined functionalities, spontaneous electron transfer, and formed a heterojunction barrier at the interface, among other things.^79,80^ These benefits led to the exploration of several 2D transition metal dichalcogenides (TMDs) and their MXene-based 2D/2D hybrids for NO_2_ gas sensing applications. Due to their high mechanical characteristics, excellent response for redox reactions, huge number of active sites, outstanding adsorption capabilities, and tuneable layer-dependent features, 2D TMDs have recently attracted interest for gas sensing applications.^81,82^ Among all the TMDs, WS_2_ and its hybrids have emerged as the most promising candidate for NO_2_ gas sensing application because of their several advantages such as tuneable band structure, low cost, high surface area, electron mobility (234 cm^2^ V^−1^ s^−1^) and ambipolar field modulation behaviour.^83,84^ The target gases such as NO_2_ can easily diffuse between the layers of WS_2_-MXene hybrids because W–S atoms in the 2D WS_2_ are covalently bonded with weak van der Waals forces between the layers. Quan *et al.* for the first time reported a paper-based NO_2_ sensor using the Ti_3_C_2_T_*x*_/WS_2_ heterostructure whose response (15.2%) was 3.2 and 76 times higher than that of the Au interdigital electrode integrated with the Ti_3_C_2_T_*x*_/WS_2_ sensor (4.8%) and Ti_3_C_2_T_*x*_ sensor (0.2%), respectively.^85^ The sensor exhibited excellent stability even under high humid conditions with a limit of detection of 11 ppb for NO_2_ gas. The NO_2_ sensing process is explained in terms of band bending in heterojunctions and changes in the width of the depletion layer upon exposure to gas molecules (Fig. 8). Free electrons migrate from WS_2_ to Ti_3_C_2_T_*x*_ to balance the Fermi level position at equilibrium, and the adsorbed oxygen ions (O_2_^−^) distribute across the material's surface, resulting in the production of a hole accumulation layer. Because NO_2_ has a larger electron affinity (2.30 eV) than O_2_ (0.44 eV), electrons migrate towards NO_2_ from NO_3_^−^ in the final step of the reaction. *i.e.*

8 2NO_2(g)_ + O_2_^−^ + e^−^ → 2NO_3_^−^

DFT calculations confirmed that the improved NO_2_ sensing performance is credited to the work function matching, heterojunction regulation effect and suppression of the metal-induced gas states. The enhanced visible light photo-activation effects, optoelectronic properties along with the efficient separation of photo carriers by the 2D/2D heterointerface of Ti_3_C_2_T_*x*_/WS_2_ helped to achieve enhanced NO_2_ sensing performance with full reversibility, good selectivity, fast response/recovery rate, long stability and low limit of detection (10 ppb).^86^

**Fig. 9:**
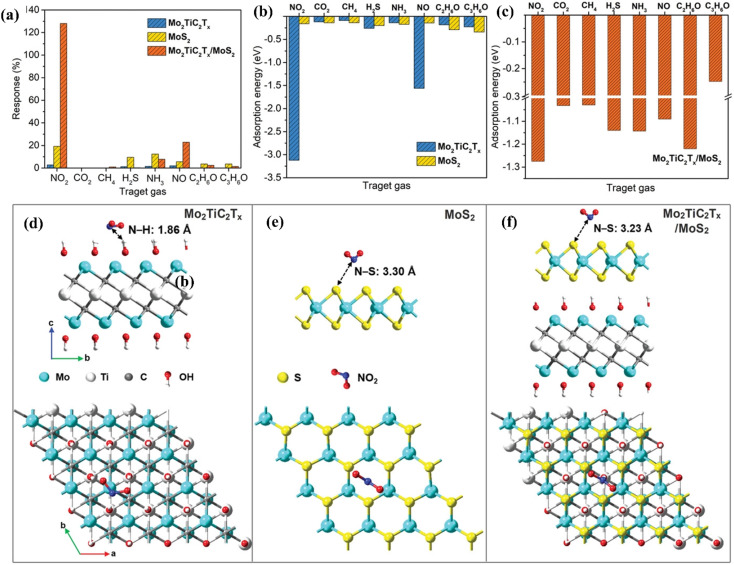
(a) Selective responses for the Mo_2_TiC_2_T_*x*_ (MXene), pristine MoS_2_, and Mo_2_TiC_2_T_*x*_/MoS_2_-based sensors in the presence of different gases. (b) Adsorption energies of pristine Mo_2_TiC_2_T_*x*_ and pristine MoS_2_ for different gases. (c) Adsorption energies of the Mo_2_TiC_2_T_*x*_/MoS_2_ heterostructure for different gases. Top and side views of the configurations for (d) Mo_2_TiC_2_T_*x*_ (e) MoS_2_, and (f) Mo_2_TiC_2_T_*x*_/MoS_2_ heterostructure after adsorption of NO_2_ molecules, reprinted in part permission from ref. 21 from Copyright (2023) Wiley materials.

MoS_2_/MXene heterostructures with interconnected networks exhibited highly sensitive and selective NO_2_ sensing properties due to the presence of abundant Mo active sites, excellent heterointerface contacts and accelerated electrons from the conductive MXene.^18,19^ The excellent response (65.6%) of the 2H MoS_2_/Ti_3_C_2_T_*x*_ MXene heterostructure to 100 ppm NO_2_ at ambient temperature is ascribed to the quick channels for carrier transportation and a large number of active sites between 2H MoS_2_ and few layered MXenes.^19^ For a 2D/2D/2D composite made up of Ti_3_C_2_T_*x*_ MXene@TiO_2_@MoS_2_, the strong interfacial contact between the different components facilitated the charge carrier transfer and spatial separation, resulting in improved sensing performance, with Ti_3_C_2_T_*x*_ and MoS_2_ acting as the electron reservoir and main sensitive materials, respectively.^20^ This NO_2_ sensor based on Ti_3_C_2_T_*x*_ MXene@TiO_2_@MoS_2_ exhibited good response (*R*_a_/*R*_g_ = 55.6 for 50 ppm NO_2_) which was 3.8, 7.3 and 2.1 times higher than that of TiO_2_@MoS_2,_ pristine MoS_2_ and MXene@MoS_2_ composites respectively. The highly active double transition metal titanium molybdenum carbide (Mo_2_TiC_2_T_*x*_) and its hybrids with MoS_2_ displayed exceptional responsiveness due to their extremely strong surface adsorption (−3.12 eV) for the gas NO_2_.^21^ The fabricated Mo_2_TiC_2_T_*x*_/MoS_2_ sensor exhibited a sensitivity of around 7.36% ppm^−1^, detection limit of 2.5 ppm and room temperature reversibility (Fig. 9). The edge-enriched Mo_2_TiC_2_T_*x*_/MoS_2_ heterostructure is thought to play a crucial role in improving the sensor performance, as both the pristine Mo_2_TiC_2_T_*x*_ and its MoS_2_ composite displayed p-type behaviour.

#### 3.2.3. MXene-other 2D material hybrids

**Fig. 10:**
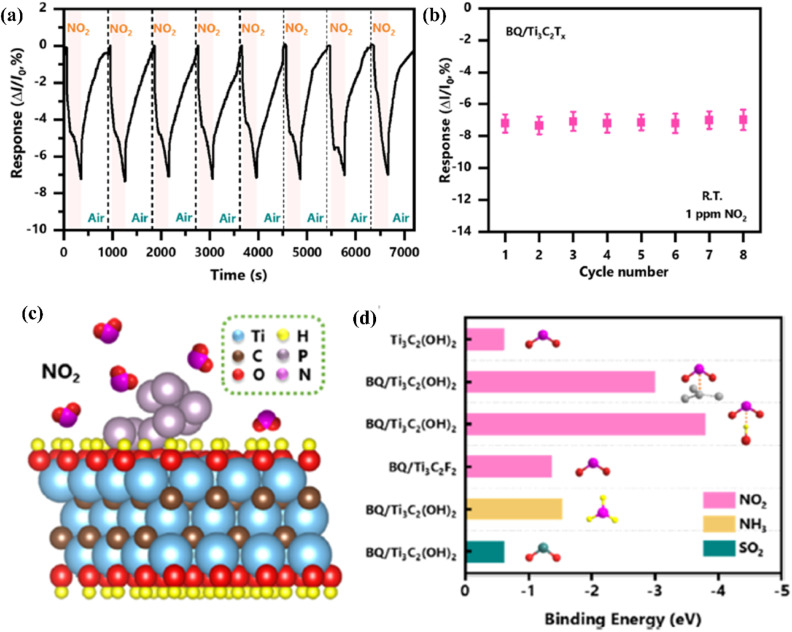
Sensing properties of Ti_3_C_2_T_*x*_ (MXene) modified with black phosphorus quantum dots in the presence of NO_2_ (a) cycling test and (b) the summarized responses of the BQ/Ti_3_C_2_T_*x*_ (MXene) sensor to NO_2_ (1 ppm). (c) Schematic representation of adsorption of NO_2_ molecules on the BQ/Ti_3_C_2_T_*x*_ (MXene) heterostructure surface. (d) Binding energies calculated of gas molecules on the pristine Ti_3_C_2_T_*x*_ (MXene) and BQ/Ti_3_C_2_T_*x*_ (MXene) heterostructure with different binding modes and functional groups, reprinted with reprinted in part permission from ref. 85 from Copyright (2023) Elsevier.

Among the other 2D materials, black phosphorus (BP) has gained much interest for gas sensing applications due to its anisotropic electrical properties, p-type conductivity with a direct band gap and excellent gas adsorption properties.^87–89^ However, its low humidity stability precludes its use in gas detection. In this context, a field effect transistor (FET) based on BP quantum dots loaded on MXene sheets is being studied for NO_2_ sensing applications due to its greater affinity for NO_2_ adsorption.^90^ The hybrid material showed a wide linear detection range from 50 ppb to 10 ppm, and an LOD of 13 ppb with 3 times higher sensitivity when compared to pristine MXene with improved selectivity and stability (Fig. 10).

#### 3.2.4. MXene-polymers and other hybrids

**Fig. 11:**
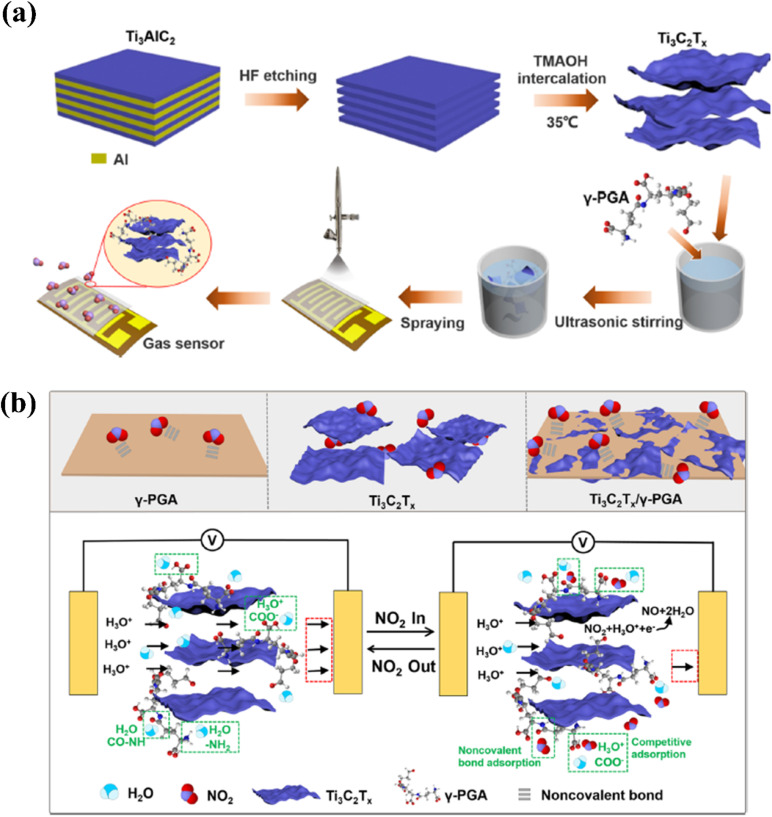
Techniques used to improve the NO_2_ sensing performance of Ti_3_C_2_T_*x*_ by modifying with γ-poly(l-glutamic acid): (a) schematic representation of the fabrication of oligo-layer Ti_3_C_2_T_*x*_ and deposition of the Ti_3_C_2_T_*x*_/γ-PGA nanocomposite film. (b) Gas sensor's NO_2_ gas-sensing mechanism, reprinted with reprinted in part permission from ref. 51 from Copyright (2023) American Chemical Society.

Polymers and other chemical functional groups have been employed to modify MXenes to improve their stability, enhance the effective adsorption and blocking effects, *etc.* for gas sensing applications.^51,91,93–95^ Naveen Kumar *et al.* found that covalently modifying MXenes (Nb_2_CT_*x*_) with (3-aminopropyl)triethoxysilane (APTES) *via* silylation molecules increased the stability and allowed for the simultaneous inclusion of extra reactive NH_2_ group.^65^ The APTES reduces the MXene oxidation by producing a homogenous, thick protective layer over the surface of Nb_2_CT_*x*_. APTES served as an electron acceptor, facilitating electron transfer to NO_2_ molecules *via* MXenes. The Nb_2_CT_*x*_-0.2 APTES MXene-based gas sensor exhibited a good sensing response (2.5 times greater than pristine MXene) with stability for more than 45 days. Owing to its electronegativity, abundant functional groups and blocking effect, γ-poly (l-glutamic acid) (γ-PGA) is used to stimulate the positive response behaviour of the Ti_3_C_2_T_*x*_ MXene-based sensor.^96^ The sensing performance of MXene/γ-PGA-based sensors was improved by 85 times (1127.3%) that of Ti_3_C_2_T_*x*_ MXene (13.2%). Further, the sensor exhibited a faster response/recovery time (43.4 s/3 s) compared to the pristine MXene-based sensor (18.5 min/18.3 min) with excellent reversibility and repeatability. Fig. 11 shows the schematic illustration of the fabrication process and the sensing mechanisms of the MXene/γ-PGA sensor. Hassan *et al.* used embedded MXene-PEDOT-PSS as a reverse side layer and as the Joule heater for the printed CNT-graphene-based NO_2_ sensor which was capable of detecting 1 ppm NO_2_ gas at 65 °C with high selectivity.^91^

### 3.3. MXene-derived materials

**Fig. 12:**
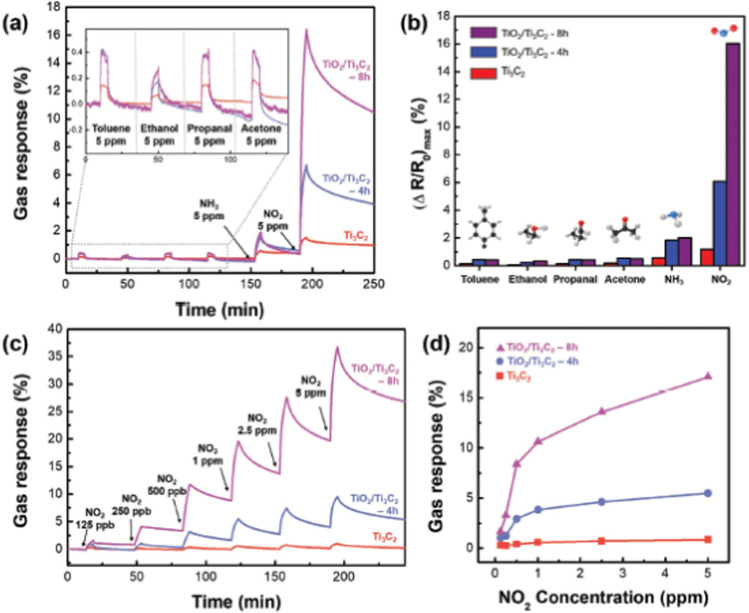
(a) Selective response to gas and (b) maximum change in resistance when exposed to 5 ppm of toluene, ethanol, propanol, acetone, NH_3_^+^, and NO_2_. (c) The TiO_2_/Ti_3_C_2_ real-time gas response curve as a function of NO_2_ concentration and (d) graph showing maximal resistance change dot at room temperature (NO_2_, at concentrations ranging from 0.125 to 5 ppm), reprinted in part permission from ref. 93 from Copyright (2023) Wiley materials.

Ti_3_C_2_ MXene-derived semiconducting–metallic Ti_3_C_2_–TiO_2_ hybrid materials are reported to show selective NO_2_ sensing properties due to the analyte surface charge transfer and the modulation of Schottky barrier (SB) at the interface between the semiconducting and metallic surfaces.^97^ The TiO_2_/Ti_3_C_2_ composite-based sensors showed selective NO_2_ sensing performance with excellent sensitivity around 13.7 times greater than pristine Ti_3_C_2_ MXene and a limit of detection of 125 ppb (Fig. 12). Similarly, in another study, Liu *et al.* reported that the composite with an optimal ratio of Ti_3_C_2_–TiO_2_ showed higher response values (86 times) and faster recovery and response times (3.8 and 2 times) to 100 ppm NO_2_ as compared to the pristine Ti_3_C_2_ MXene.^98^ Theoretical calculations proved that the two Ti–O bonds and the development of a Ti–N bond between the N and O atoms from the NO_2_ and the nearby Ti atoms from the Ti_3_C_2_ in the composites result in a substantial rise in the adsorption energy. Song *et al.* reported a high-performance NO_2_ sensor based on MXene-derived TiO_2_ nanoparticle intercalated between reduced graphene oxide (rGO) assembly in which uniform distribution of the TiO_2_ nanoparticles and highly wrinkled rGO interconnected porous structure contributed to the sensing.^99^ The MXene-derived TiO_2_ spaced rGO gas sensor exhibited a 400% enhancement in NO_2_ sensitivity with a limit of detection of around 50 ppb, excellent workability under humid conditions and good selectivity. Further, SnS_2_-MXene-derived TiO_2_ hybrid materials exhibited a large response of 115 against 1000 ppm NO_2_ gas with an ultrafast recovery time of 10 s at room temperature.^100^

## 4. Conclusions and future directions

In this review article, we highlighted the state-of-the-art use of MXene-based materials for room temperature NO_2_ sensing applications. The advantages of MXenes such as their high surface area to volume ratio, tuneable physicochemical properties, high metallic characteristics, hydrophilicity, mechanical flexibility and availability of abundant surface functional groups make them ideal candidates for the fabrication of high performance NO_2_ sensor applications. Subsequently, we discussed the NO_2_ sensing mechanisms of pristine and heterostructures of MXenes in detail. Among the various reported MXenes, Ti_3_C_2_T_*x*_ is the most explored one for NH_3_ and VOC sensing. However, it possesses small adsorption energy (>−0.8 eV) for NO_2_ gas suggesting its limited selectivity. The strong interlayer van der Waals force of attraction lead to the self-stacking of MXene nanosheets leading to the obstruction in the diffusion pathways and insufficient utilization of surface active sites, thereby reducing gas-sensing response. NO_2_ molecules capture electrons from the Ti_3_C_2_T_*x*_ to produce NO_2_^−^ and oxidize Ti_3_C_2_T_*x*_ that leads to poor reversibility. Due to these demerits, the sensing performance of other MXenes such as Mo_2_CT_*x*_ and V_2_CT_*x*_ are also investigated, and it was discovered that these MXenes have great potential in NO_2_ gas sensing applications, which opens up the door for unexplored MXenes for NO_2_ sensing applications. The literature studies prove that the metallic conductivity of MXenes along with interlayer spacing is responsible for positive change in resistance.

The strategies such as introducing interlayer spacers (*e.g.*, TiO_2_), constructing self-supporting architectures and hybrid formation with other semiconducting materials are adopted to overcome the self-restacking problem of MXenes and thereby enhancing the adsorption site. From the literature, we can see that MXene hybrid formation with different metal oxides, TMDs, black phosphorus, polymers, *etc.* among others helps in the fabrication of MXene-based high-performance NO_2_ sensors with high response, selectivity, low response and recovery time, *etc.* The sensor's response value and response speed are improved here due to the sufficient and compact interface contact, which can promote interfacial charge transfer. Furthermore, the *in situ* formed heterogeneous composite shows potential in gas sensor applications. This *in situ* heterogeneous composite production incorporates the structural benefits of multi-component materials while preventing the self-stacking of MXene nanosheets.

**Fig. 13:**
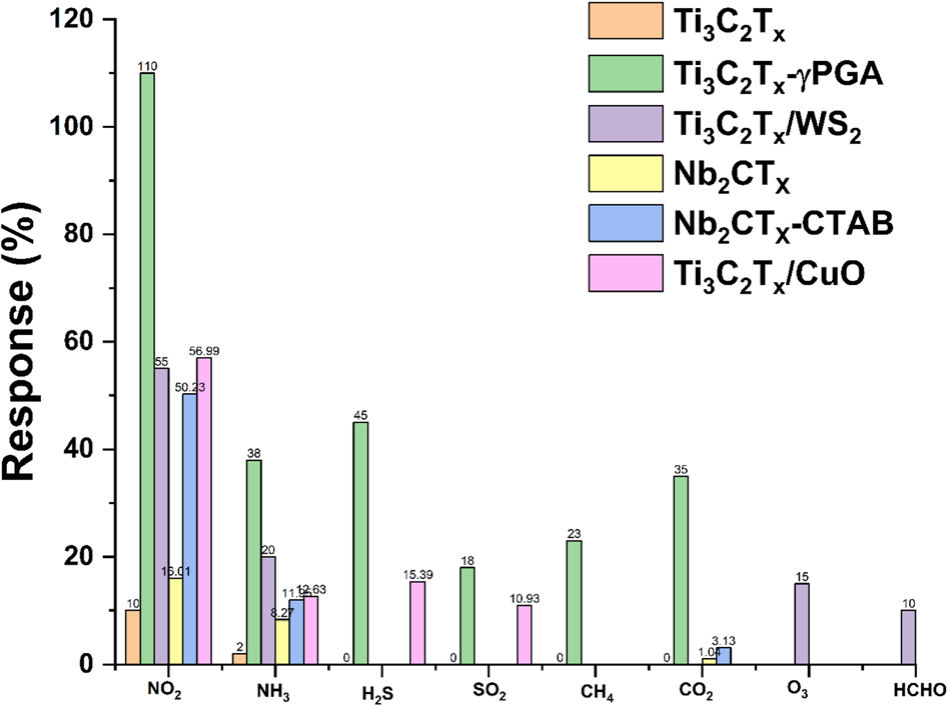
Selective nature of MXenes towards NO_2_ as compared to other gases.

However, although there are reports on these materials for NO_2_ sensing still there is plenty of room in this topic which need to be explored for the design of high performance NO_2_ sensors. At this point, research on monolayer MXenes in gas sensors is in its early stage, despite the fact that Choi *et al.* revealed that monolayer Mo_2_CT_*x*_ performs better against NO_2_ but performs poorly without intercalants. Thus, the sensing capability of monolayer MXenes falls short of the criteria for practical applications, specifically in terms of its sensitivity and low applicability under ambient conditions.^53^ This can be clearly seen from Fig. 13, where the pristine MXene shows less response towards NO_2_ and its modifications with polymers help to enhance its sensitivity towards NO_2_. Several newly reported MXenes are yet to be explored for the fabrication of high performance NO_2_ sensor devices.^25^ Similarly, heterostructures of these newly emerging MXenes with other 2D materials such as TMDs, BP, MBenes, *etc.* are less explored. Hence, choosing the appropriate combination of materials with MXenes for the fabrication of heterostructures and tuning their properties by defect and vacancy engineering, alloying, doping, intercalation, layer tuning, *etc.* can allow for high performance selective NO_2_ gas sensing performance.

Aside from sensor recovery and response time, the immediate response of the gas sensor is an important aspect. The response time of each sensor is determined by how quickly the gas molecules react to the sensing film and change their corresponding parameter. So far, the observed response time of NO_2_ molecule detection by MXene's has been in the few seconds' range. As a result, developing NO_2_ sensors capable of responding in milliseconds or microseconds remains difficult, and only one study is now available.^69^ The technique for improving ultrafast sensors is based mostly on the interaction of gas molecules and MXenes as well as charge transfer in MXenes. By developing MXene-based heterostructures as sensing devices, the rapid charge carrier separation can be increased. Different methods such as photoexcitation, doping, gating, defect and vacancy engineering, surface modification, piezotronic/piezophotonic effects, *etc.* for the MXene-based NO_2_ sensor devices are yet to be explored.

Recently, it is revealed that 2D material-based gas sensors with Schottky contact can create highly selective and sensitive sensors by adjusting the Schottky barrier height (SBH), which works as the gate controller to regulate the current flow. As a result of the metallic character of MXenes, SBH-controlled high-performance NO_2_ selective sensors can be produced by selecting appropriate semiconducting materials for Schottky contact creation.^101–104^ It is also necessary to strengthen theoretical and experimental efforts with thorough insight and understanding, which will lead to the advancement of high-performance NO_2_ sensors. Several pathways for developing high-performing electrical contacts also should be identified.

Environmental factors include contaminants of different chemicals, humidity, moisture, corrosion caused by toxic vapours, and residual charges all have a substantial impact on a film's conductivity. These factors significantly lower the gas sensors' stability, reliability and repeatability. The response of MXene-based RT NO_2_ sensors has been found to decrease with an increase in humidity. This is due to the interaction between adsorbed oxygen O_2_^−^ and water molecules that led to decreasing the site required for the adsorption of NO_2_ molecules.^68^ So, various approaches for reducing humidity interference such as (i) pre-treating the target gas such as NO_2_ using dehumidifier (ii) modifying materials using hydrophobic materials and (iii) establishing a humidity compensation model are frequently utilized. Zhao *et al.* demonstrated the humidity compensation model can be established for NO_2_ gas sensing at a concentration of 2–10 ppm. They also applied statistical regression to find the relationship between gas concentration, humidity and gas sensing response (*R*). The sensor based on MXene/γ-PGA was able to recognize NO_2_ with 2 ppm concentration after humidity compensations. Thus, it is necessary to make efforts to improve the sensing devices' stability and response.^92^

According to WHO, the recommended levels of NO_2_ exposure for an hour are 82 ppb and for a year are 410 ppb. Long-term exposure to NO_2_ above that threshold has negative health effects. The MXene-based NO_2_ sensor's lower detection limit has been measured in ppb. Consequently, it takes a lot of work to produce ultrasensitive NO_2_ sensors, which is an important task. Finding NO_2_-sensitive materials that can quickly and easily integrate with MXenes and quickly detect NO_2_ at lower concentrations is crucial. Additionally, for quick sensor response, such materials need to speed up the transfer of charges.

Also, considering the advantages of 2D MXenes, flexible and wearable sensor devices based on these materials need to be explored. Till now there are only two reports on self-powered NO_2_ sensors based on MXenes which displayed promising features over conventional gas sensor devices. Hence, this research topic is believed to be an emerging research area in recent years.

Spectroscopic techniques that use electrical shields and laser sources have recently caught the attention of scientists for NO_2_ detection at trace quantity. The visible range of the absorption spectrum of NO_2_ molecules provides a significant opportunity for electronic exciton in NO_2_ molecules. Using spectroscopic methods for NO_2_ trace detection with MXene sensors could be a novel strategy. Over the past two years, the scientific community has become interested in light-assisted sensing of NO_2_ gas molecules. The MXene's ability to combine with metal layers has gained a lot of attention in surface plasma resonance (SPR) sensors.^105^ Thus, the SPR properties of MXenes may be an unconventional approach to construct NO_2_ gas sensors based on MXenes. New experimental attempts should be directed towards realising the potential of plasmonic in the field of gas sensing. SPR can activate the interface between MXene and metal, changing the refractive index. Thus, a good selection of metal NPs and appropriate wavelengths will aid in the development of high-performance NO_2_ gas sensors.

**Fig. 14:**
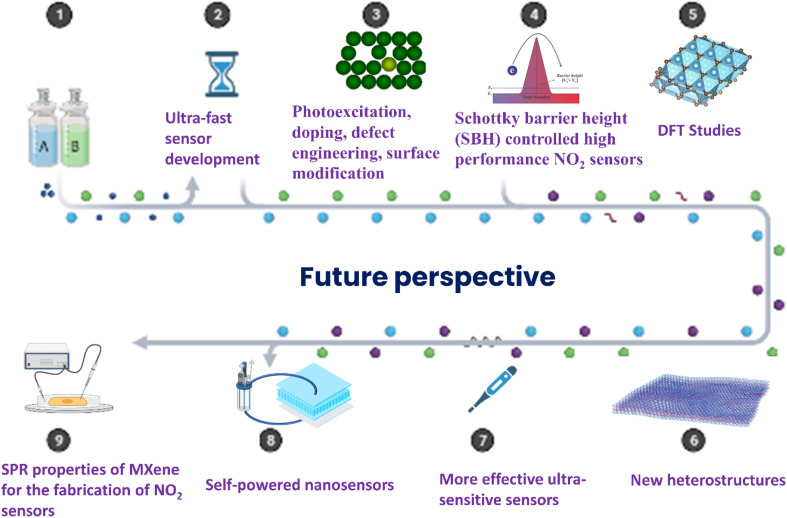
Future perspectives of MXene-based NO_2_ gas sensors, reprinted with permission from, *Sci. Rep.*, **9**, https://doi.org/10.1038/s41598-019-45162-7 and http://biorender.com/.

By considering these aspects, it can be concluded that MXenes are still in the initial phase of gas sensor research and further exploration into these topics is needed to achieve high performance NO_2_ sensor devices (Fig. 14). Understanding the NO_2_ gas sensing mechanisms of MXene-based materials by different *in situ*/operando spectroscopic studies and by detailed theoretical investigations is of significant importance since it is helpful to design high performance gas sensors.^37,106–109^ These studies can provide detailed information on the physical and chemical properties of the MXenes upon interaction with the analyte gas molecules. But there is no report on these topics to date. As a result, these study domains must be investigated in the next few years in order to develop superior gas sensor devices.

## Conflicts of interest

There are no conflicts to declare.

## Supplementary Material
